# Author Correction: Understanding the plume dynamics of explosive super-eruptions

**DOI:** 10.1038/s41467-018-05545-2

**Published:** 2018-08-22

**Authors:** Antonio Costa, Yujiro J. Suzuki, Takehiro Koyaguchi

**Affiliations:** 1 Istituto Nazionale di Geofisica e Vulcanologia, 40128 Bologna, Italy; 2 Earthquake Research Institute, University of Tokyo, 1-1-1 Yayoi, Bunkyo-ku, Tokyo, 113-0032 Japan

Correction to: *Nature Communications* 10.1038/s41467-018-02901-0; published online 12 Feb 2018

We became aware of a mistake in the data displayed in the original version of Fig. 3. Specifically, the lines showing the relationship between column height and MFR for MFR larger than 10^10^ kg/s were based on simulations in which the exit gas fraction was assumed to be an unrealistic value of 0.76 rather than the correct value of 0.33. The correct version of Fig. 3 is:

**Figure 1:**
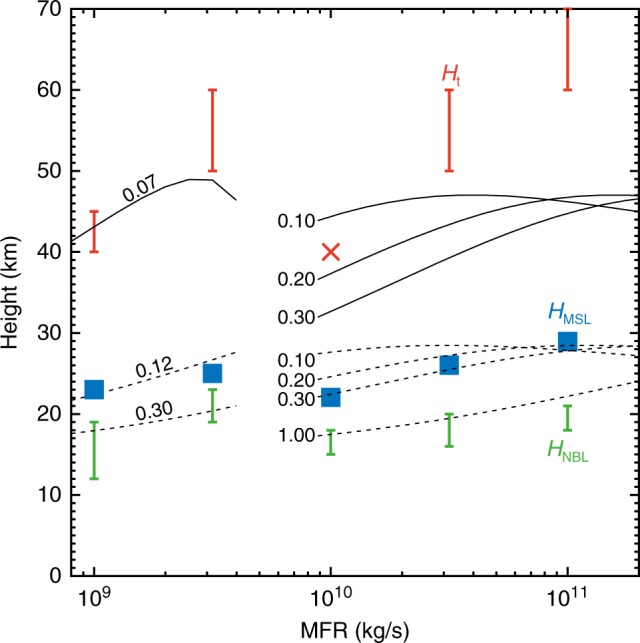
Fig. 3

which replaces the previous incorrect version:  This has been corrected in both the PDF and the HTML versions of the Article. The text was written on the basis of the correct plots, and so this error does not affect the original discussion or conclusions of the Article. The authors apologize for the confusion caused by this mistake.

