# Supplementary material for: Understanding the plume dynamics of explosive super-eruptions
Source: Nat Commun. 2018 Feb 13;9:654. doi: 10.1038/s41467-018-02901-0 (PMC5811558; doi:10.1038/s41467-018-02901-0)
Supplement: Supplementary file 1 — Supplementary Information [file 41467_2018_2901_MOESM1_ESM.pdf]

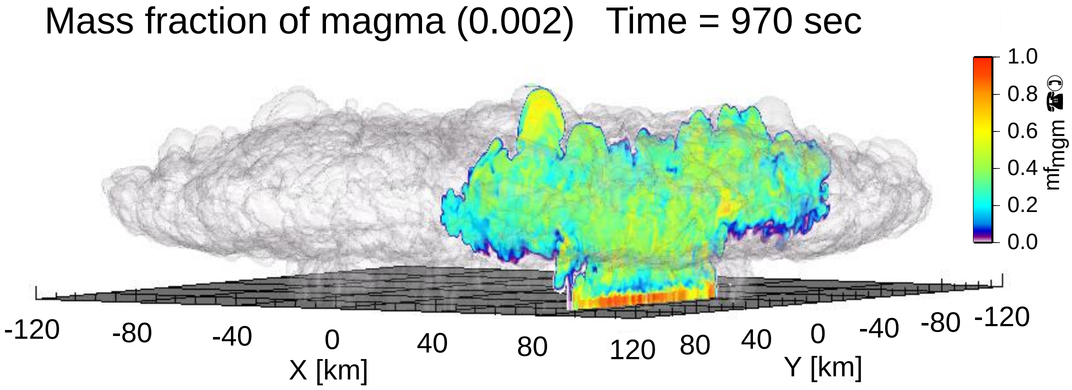

Supplementary Figure 1 Visualization of the development of local super-plumes above the main umbrella region for extremely large MFR (10^{11} kg/s). The colour scale indicates the mass fraction of the erupted mixture mf_{mgm}.

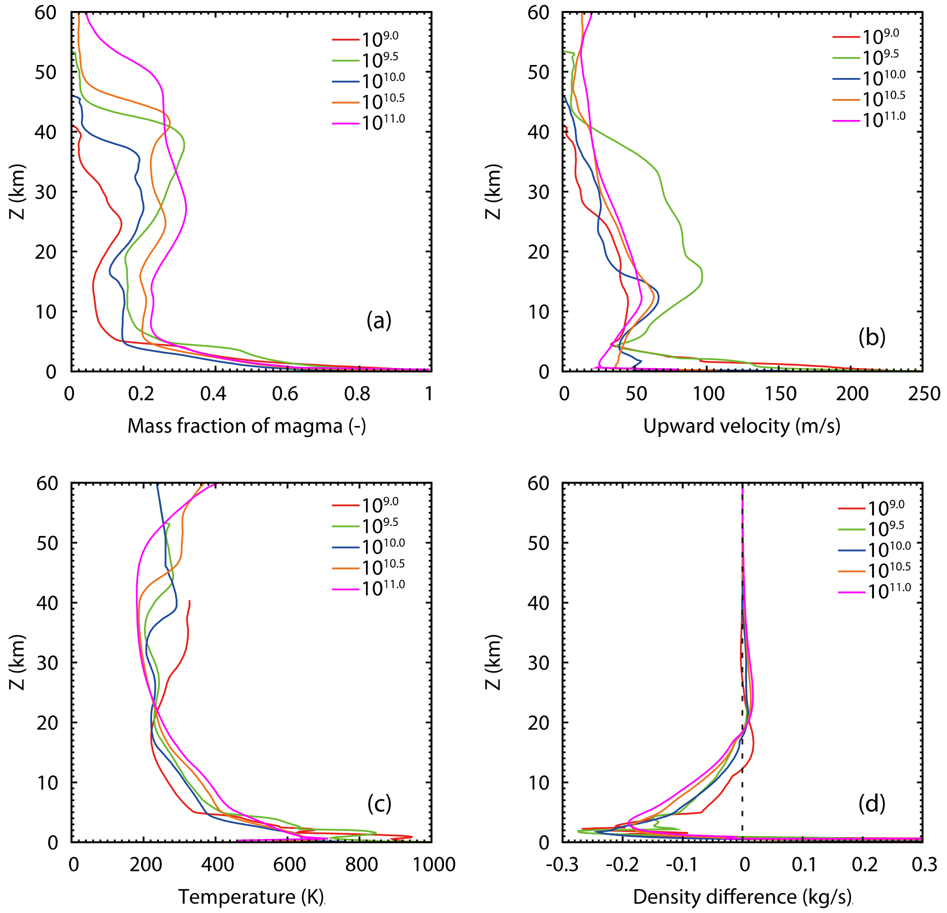

Supplementary Figure 2 Variation with height of the average variables of the 3D simulation results for the different MFR, calculated as in Cerminara et al (2016): a) mass fraction, b) vertical velocity, c) temperature, d) density difference with respect to the atmosphere.

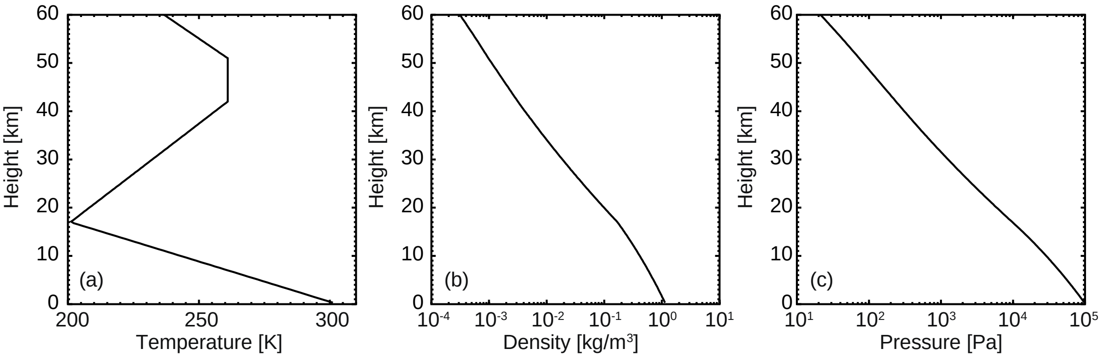

Supplementary Figure 3 Atmospheric profiles used for the simulations representing typical tropical conditions[23].

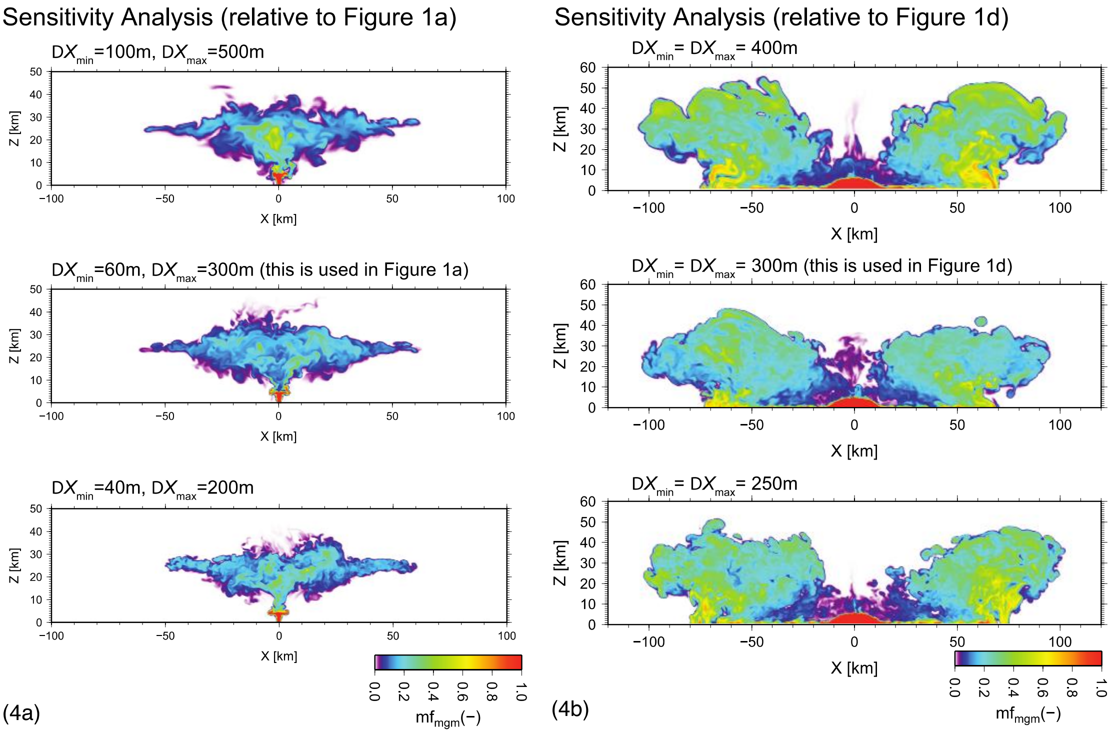

Supplementary Figure 4 Sensitivity analysis on the effects of the computational grid for the two representative cases reported in Figure 1a (4a) and Figure 1d (4b).
