# Supplementary material for: Understanding the plume dynamics of explosive super-eruptions
Source: Nat Commun. 2018 Feb 13;9:654. doi: 10.1038/s41467-018-02901-0 (PMC5811558; doi:10.1038/s41467-018-02901-0)
Supplement: Supplementary file 3 — Description of Additional Supplementary Files [file 41467_2018_2901_MOESM3_ESM.docx]

**Description of Additional Supplementary Files**

File Name: Supplementary Movie 1

Description: Time evolution of different variables for MFR=10^9^ kg/s from the starting of the eruption up to 3600 sec, for a circular vent through vertical cross-sections of mass fraction, density difference, radial velocity, and vertical velocity (left side); three dimensional isosurface and top view where the mass fraction of the erupted mixture is 0.01 (right side).

File Name: Supplementary Movie 2

Description: Time evolution of different variables for MFR=10^9.5^ kg/s from the starting of the eruption up to 3600 sec, for a circular vent through vertical cross-sections of mass fraction, density difference, radial velocity, and vertical velocity (left side); three dimensional isosurface and top view where the mass fraction of the erupted mixture is 0.01 (right side).

File Name: Supplementary Movie 3

Description: Time evolution of different variables for MFR=10^10^ kg/s from the starting of the eruption up to 3600 sec, for a circular vent through vertical cross-sections of mass fraction, density difference, radial velocity, and vertical velocity (left side); three dimensional isosurface and top view where the mass fraction of the erupted mixture is 0.01 (right side)

File Name: Supplementary Movie 4

Description: Time evolution of different variables for MFR=10^11^ kg/s from the starting of the eruption up to 970 sec, for a circular vent through vertical cross-sections of mass fraction, density difference, radial velocity, and vertical velocity (left side); three dimensional isosurface and top view where the mass fraction of the erupted mixture is 0.01 (right side).

File Name: Supplementary Movie 5

Description: Time evolution of different variables for MFR=10^11^ kg/s from the starting of the eruption up to 3600 sec, for a circular vent through vertical cross-sections of mass fraction, density difference, radial velocity, and vertical velocity (left side); three dimensional isosurface and top view where the mass fraction of the erupted mixture is 0.01 (right side).

File Name: Supplementary Movie 6

Description: Time evolution of different variables for MFR=10^11^ kg/s from the starting of the eruption up to 1000 sec, for a fissure (20 km × 0.5 km) vent through vertical cross-sections of mass fraction, density difference, radial velocity, and vertical velocity (left side); three dimensional isosurface and top view where the mass fraction of the erupted mixture is 0.01 (right side).
